# Fat Embolism: A Rare Complication of Bone Biopsy

**DOI:** 10.7759/cureus.44765

**Published:** 2023-09-06

**Authors:** Daniela Madeira, Ana Orfão, Clara Matos, Patrícia Vasconcelos

**Affiliations:** 1 Internal Medicine, Hospital Professor Doutor Fernando Fonseca, Amadora, PRT

**Keywords:** drug reaction with eosinophilia and systemic symptoms (dress), b cell lymphoma, plasma free fatty acids, bone marrow, bone biopsy, fat embolism, iatrogenesis

## Abstract

We report a woman who was admitted to the hospital with a sudden onset of extensive maculopapular erythematous rash involving the trunk and extremities, six weeks after initiating antihypertensive medication. She had atypical lymphocytosis with *Gumprecht* shadows, elevated liver enzymes, and acute kidney injury. The diagnosis of drug reaction with eosinophilia and systemic symptoms (DRESS) syndrome secondary to antihypertensive drugs was suspected and the antihypertensive drugs were suspended. A hypothesis of lymphoproliferative disease was also considered, and consequently, a myelogram and bone biopsy of the iliac crest were performed. After the procedure, the patient developed acute hypoxemia. After the exclusion of pulmonary thromboembolism by CT angiography, we assumed a presumptive diagnosis of iatrogenic fat embolism syndrome (FES) associated with bone biopsy. The patient deteriorated with worsening hypoxemia and ultimately died. This case represented a diagnostic challenge and highlighted iatrogenesis's undesirable and potentially fatal effects. Careful consideration of the risk-benefit ratio of all medical procedures is paramount in daily medical practice and knowledge of the possible risks is necessary for their early recognition and therapeutic approach.

## Introduction

According to the World Health Organization, iatrogenesis corresponds to a deleterious, undesirable, and unintentional effect of a medical act. It is the fifth leading cause of death worldwide and is considered a major threat to health [1]. The medical act includes the actions we take as physicians during our professional practice, involving everything we do while seeing our patients. This is not only about scientific knowledge but also about how we behave when dealing with our patients and their parents. The acts may involve seeing a patient, selecting medication and providing therapeutic indications, performing a procedure, or making a wrong diagnosis, and others [1,2].

## Case presentation

 An African and autonomous 89-year-old woman with arterial hypertension and dyslipidemia was admitted to our hospital with rounded and small maculopapular eruptions with follicular accentuation that evolved into an erythematous rash involving the trunk and extremities, which began six weeks after starting to take perindopril, amlodipine, and hydrochlorothiazide.

Laboratory tests revealed atypical lymphocytosis with Gumprecht shadows (leucocytes 15.900/µL: neutrophils 7.900/µL (49.9%), lymphocytes 6.700/µL (42.4%), monocytes 1.000/µL (6.3%), eosinophils 200/µL (1.1%), basophils 100/µL (0.3%)), hemoglobin 11.7g/dL, platelets 364.000/µL, elevated sedimentation rate (104mm), C-reactive protein 2.63mg/dL; elevated liver enzymes (aspartate aminotransferase 389U/L; alanine aminotransferase 616U/L; alkaline phosphatase 1367U/L; gamma-glutamyltransferase 1,131UI/L; total bilirubin 11mg/dL), and acute kidney injury (creatinine 4mg/dL), without ionic abnormalities. Cervical-thoracic-abdominal-pelvic computed tomography (CT) was performed and did not reveal any changes. Considering the skin changes and concomitant organic dysfunction, the hypothesis of drug reaction with eosinophilia and systemic symptom (DRESS) was raised. We applied the RegiSCAR score for DRESS probability and admitted a possible case of DRESS (2 points), secondary to the antihypertensive medication.

**Figure 1:**
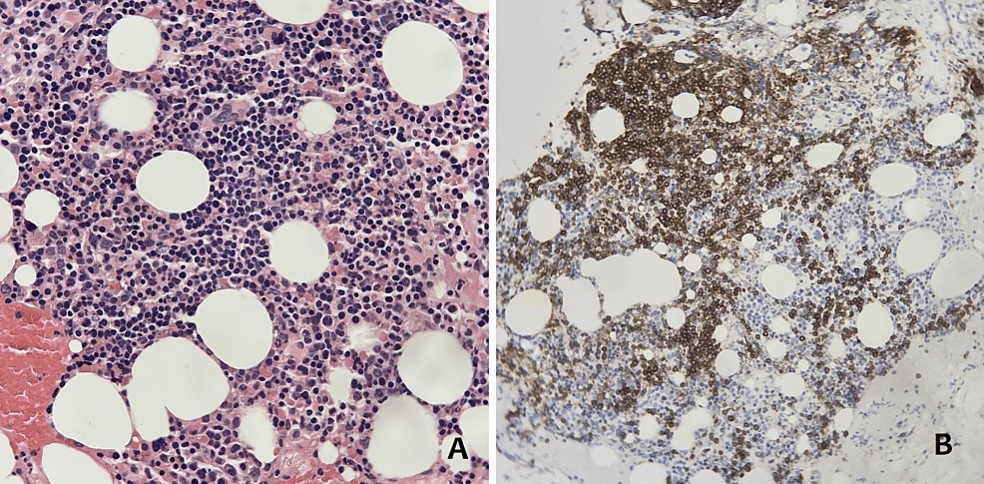
Bone marrow aspiration and biopsy of the iliac crest (A) Hematoxylin and eosin stain. (B) Immunohistochemistry showing interstitial infiltration by small, CD20-positive lymphocytes, compatible with non-Hodgkin B lymphoma.

Given the presence of lymphocytes with Gumprecht shadows on the blood smear, which is a finding sometimes associated with lymphoproliferative disorders, immunophenotyping of B cells was performed and the patient was submitted to a bone marrow aspirate and biopsy of the iliac crest, whose results were suggestive of a low-grade B-cell lymphoma (Figures 1A, 1B). Immunophenotyping was negative for CD-5 and CD-10.

**Figure 2:**
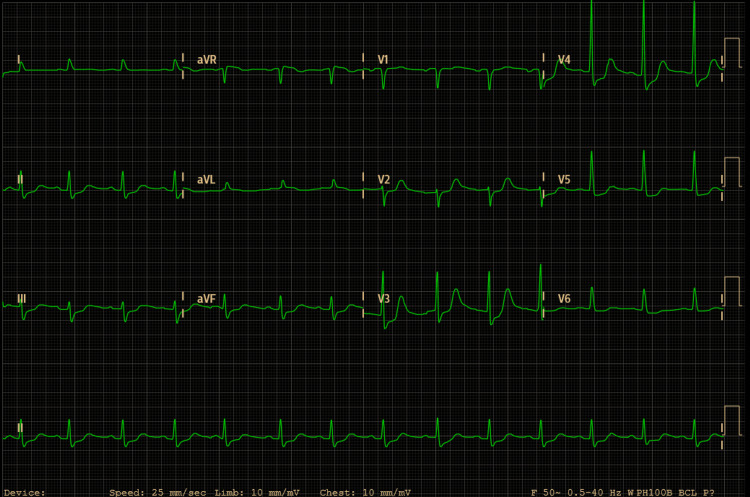
Electrocardiogram showing alteration of ventricular repolarization

**Figure 3:**
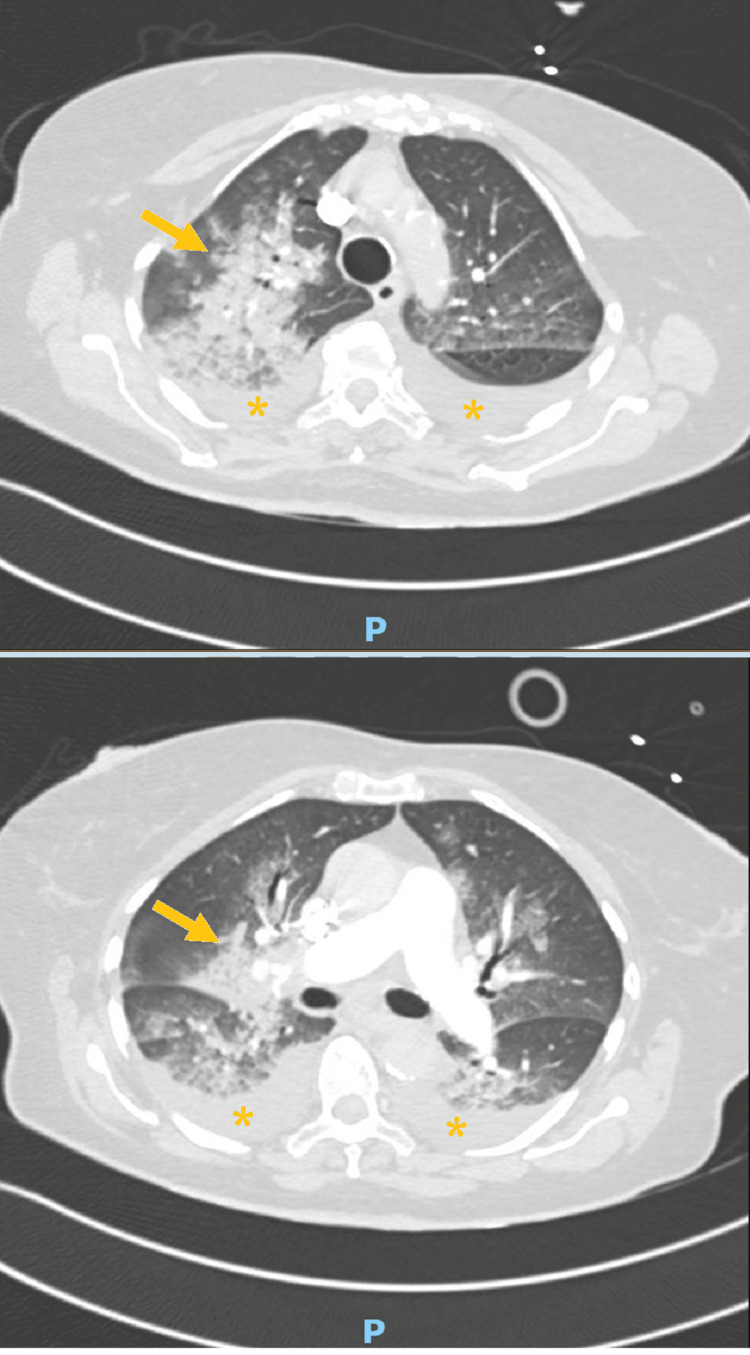
Chest computed tomography angiography Arrows: consolidation in the apical segment of the right upper lobe. Asterisks: minor bilateral pleural effusion.

The day after the procedure, she developed severe hypoxemia and an altered state of consciousness. The blood tests revealed cardiac biomarkers elevation (high sensitivity T troponin 1,512ng/L), without an increase in inflammatory parameters, the electrocardiogram (Figure 2) presented alteration of ventricular repolarization in all leads, and the echocardiogram only documented mild hypokinesia of the inferolateral wall. A chest CT angiography was performed, excluding pulmonary thromboembolism (PTE), and revealed consolidation in the apical segment of the right upper lobe (Figure 3 arrows), probably related to an inflammatory process, and minor bilateral pleural effusion (Figure 3 asterisks).

Due to the absence of fever or elevation of inflammatory parameters, and concomitant taking ceftriaxone for urinary infection, we considered pneumonia less likely and admitted fat embolism syndrome (FES). Measurement of plasma-free fatty acids was increased (44mg/dL). Non-invasive ventilation (continuous positive airway pressure with expiratory positive airway pressure of 10cmH_2_O and fraction of inspired oxygen of 100%) was started with transient improvement of dyspnea; however, she has evolved to acute respiratory distress syndrome (ARDS) with severe hypoxemia (arterial blood gas test: pH 7.1; partial pressure of carbon dioxide 47mmHg; partial pressure of oxygen 43mmHg; bicarbonate 14.6 mEq/L; oxygen saturation 63%; lactate 10.13mmol/L) requiring invasive mechanical ventilation. The patient was admitted to the intensive care unit and died three days later.

## Discussion

This case is a diagnostic challenge, where we identified some possible overlapping diagnostics: DRESS, lymphoma, and FES. The former is characterized by cutaneous and hematological changes, lymphadenopathies, and organ dysfunction, in the context of a drug hypersensitivity reaction [3]. This is a rare clinical entity with variable presentation and duration [4]. The DRESS syndrome usually has a prolonged course, because there is a latency of two to eight weeks between the drug exposure and the beginning of the symptoms; six to nine weeks of recovery; and the possibility of multiple relapses. The most frequent symptoms are fever, asthenia, lymphadenopathy, and rash, which is often the first clue to the diagnosis and characterized by maculopapular eruption that may evolve into coalescing erythema [3,5]. Laboratory findings include leukocytosis with eosinophilia and/or atypical lymphocytosis. The organs that are usually involved are the liver, kidney, and lung [4,5]. The symptoms, in this case, were mild (only rash) but typical. The addition of laboratory findings (atypical lymphocytosis, renal and hepatic injury) raised suspicion of this syndrome, as it encompasses all the changes in one diagnostic. The scores applied also support this hypothesis. On the other hand, the results of the immunophenotyping and histopathology of bone marrow were suggestive of a low-grade B-cell lymphoma. Thereby we admitted the immunosuppression of the haematological disease favoured the development of DRESS because rash and organ damage are not explained only by the low-grade lymphoma. The most frequently associated drugs are antiepileptics and allopurinol; however, any drug may be involved [5]. According to the scoring system for classifying DRESS [6], this case was considered to be probably associated with an antihypertensive drug, as it appeared six weeks after its onset and improved with its suspension. The approach is based on rapid identification, drug withdrawal, and supportive measures. In patients with severe organ dysfunction, corticosteroids or cyclosporine may be considered [7]. In this case, no treatment aimed at DRESS was started at the presentation because it was essential to exclude other diagnoses, namely hematological malignancies. Mortality from DRESS is 5%-10% and it is associated with acute liver failure, fulminant myocarditis, hemophagocytosis, or multiorgan dysfunction [5,7]. FES was first described by Zenker in 1861, although pathophysiological (mechanical and biochemical) theories were only proposed in the 1920s by Gauss and Lehman and Moore [8]. This syndrome is characterized by the presence of fat emboli in the pulmonary circulation and consequent respiratory and systemic symptoms [8]. This is also a rare clinical entity, resulting from the manipulation of fatty tissue, due to orthopedic pathology, bone marrow biopsy or a transplant, plastic surgery, or extensive burns [9]. The mechanisms responsible for FES are related to the mechanical occlusion of the vessels and the inflammatory response produced by the circulation of toxic material from the emboli [9,10]. The symptoms usually begin 24-72 hours after the triggering event and are characterized by the triad of respiratory distress, neurological changes, and petechial rash. Respiratory symptoms are the most recurrent and may mimic ARDS, as described in this case [11]. Laboratory findings are non-specific and may include anemia, thrombocytopenia, changes suggestive of disseminated intravascular coagulation, and elevation of C-reactive protein [8,11]. There are no specific FES biomarkers. The measure of free fatty acids is not normally recommended due to lack of scientific evidence, however, the elevation of plasma free fatty acids in this particular case, despite not confirming the diagnosis, does support our hypothesis [8,12].

In the majority of patients, lung imaging discloses no changes; however, in some cases, there are non-specific but characteristic radiological features. Therefore, in combination with classic symptoms in the appropriate clinical setting, they can help to achieve de diagnosis. Chest radiography often shows bilateral diffuse opacities that may be indistinguishable from other illnesses, such as pneumonia, pulmonary edema or aspiration, so the usefulness of this test is limited to monitoring disease progression [12]. Chest CT is the modality of choice, as it not only suggests the diagnosis but also indicates alternative causes. This usually reveals ground-glass opacities and areas of crazy paving and, less frequently, lobar consolidations. Small centrilobular nodules, predominantly peripheral and distributed in the upper lobe, and small bilateral effusions have also been reported [11,12]. The pulmonary symptoms correlate with the extent of imaging findings [8,12]. In conclusion, there is no gold standard diagnostic test. The diagnosis is essentially clinical and made after the exclusion of other possible causes [8,9]. In this case, FES was admitted iatrogenic due to bone marrow aspirate and biopsy, considering there is bone fat tissue manipulation in these procedures the onset of symptoms was soon after that, and other diagnoses were excluded. No inflammatory parameters were augmented, so the chest image changes were associated with pulmonary inflammation and edema of FES. The exclusion of PTE and the absence of significant changes on the echocardiogram also make this diagnosis more likely. There are only a few cases like this described in the literature [13-15]. Since no targeted therapy exists, supportive treatment is the mainstay therapy while recovery is ongoing. This includes support to respiratory failure with oxygenation and noninvasive or invasive mechanical ventilation. When the patient progresses to shock, fluid resuscitation, vasopressors, mechanical cardiac support devices, or extracorporeal membrane oxygenation are recommended. Given the inflammatory response to circulating fat, some authors suggest the administration of systemic corticosteroids, although this is controversial due to the lack of trials on the subject. All the measures should be maintained until FES resolves [8-10]. Most cases are reversible and transient. Mortality rates have improved markedly with modern intensive care and range from 5% to 15%. This is typically related to severe respiratory failure and refractory shock [8,9,12]. Due to the clinical severity of multiorgan failure (respiratory and cardiac), the patient died.

## Conclusions

The present case represents a diagnostic challenge and highlights iatrogenesis's undesirable and potentially fatal effects. The prescription of new drugs was responsible for analytical changes (in the context of DRESS) that motivated more investigation and performance of complementary diagnostic tests which also had iatrogenic complications (FES).

Iatrogenicity can be associated with serious and even fatal complications, so its early identification and treatment are essential. In clinical practice, strategies may be adopted that aim to reduce their frequency, morbidity, and mortality. We suggest investigation and further testing of drugs, elaboration of protocols that assess medical errors and bad practices, and review of medical prescriptions.

## References

[1] Peer RF, Shabir N (2018). Iatrogenesis: a review on nature, extent, and distribution of healthcare hazards. J Family Med Prim Care.

[2] Cernadas JMC (2018). A broader perspective of iatrogenesis. Arch Argent Pediatr.

[3] Maja Mocjenhaupt (2023). Drug reaction with eosinophilia and systemic symptoms (DRESS). https://www.uptodate.com/contents/drug-reaction-with-eosinophilia-and-systemic-symptoms-dress#:~:text=Drug%20reaction%20with%20eosinophilia%20and%20systemic%20symptoms%20%28DRESS%29,heterogeneous%2C%20and%20the%20disease%20course%20is%20typically%20prolonged.

[4] Cacoub P, Musette P, Descamps V, Meyer O, Speirs C, Finzi L, Roujeau JC (2011). The DRESS syndrome: a literature review. Am J Med.

[5] Kardaun SH, Sekula P, Valeyrie-Allanore L (2013). Drug reaction with eosinophilia and systemic symptoms (DRESS): an original multisystem adverse drug reaction. Results from the prospective RegiSCAR study. Br J Dermatol.

[6] Chen YC, Chiu HC, Chu CY (2010). Drug reaction with eosinophilia and systemic symptoms: a retrospective study of 60 cases. Arch Dermatol.

[7] Kardaun SH, Sidoroff A, Valeyrie-Allanore L, Halevy S, Davidovici BB, Mockenhaupt M, Roujeau JC (2007). Variability in the clinical pattern of cutaneous side-effects of drugs with systemic symptoms: does a DRESS syndrome really exist?. Br J Dermatol.

[8] Rothberg DL, Makarewich CA (2019). Fat embolism and fat embolism syndrome. J Am Acad Orthop Surg.

[9] Stein PD, Yaekoub AY, Matta F, Kleerekoper M (2008). Fat embolism syndrome. Am J Med Sci.

[10] Akhtar S (2009). Fat embolism. Anesthesiol Clin.

[11] Kao SJ, Yeh DY, Chen HI (2007). Clinical and pathological features of fat embolism with acute respiratory distress syndrome. Clin Sci (Lond).

[12] Newbigin K, Souza CA, Torres C (2016). Fat embolism syndrome: state-of-the-art review focused on pulmonary imaging findings. Respir Med.

[13] Baselga J, Reich L, Doherty M, Gulati S (1991). Fat embolism syndrome following bone marrow harvesting. Bone Marrow Transplant.

[14] Onal IK, Sümer H, Tufan A, Shorbagi A (2005). Bone marrow embolism after bone marrow aspiration and biopsy. Am J Hematol.

[15] Sharma P, Gautam A, Kumar P, Malhotra P, Nada R, Ahluwalia J (2022). Bone marrow emboli following bone marrow procedure: a possible complication. Indian J Pathol Microbiol.

